# TVIR 2.0: an enhanced database of the vegetables information resources

**DOI:** 10.1093/hr/uhaf239

**Published:** 2025-09-17

**Authors:** Tong Yu, Xiao Ma, Zhuo Liu, Tongbing Su, Chenhao Zhang, Lusheng Guo, Zipeng Meng, Di Guo, Nana Yao, Yingchao Zhang, Haibin Liu, Xiaoming Song

**Affiliations:** School of Life Sciences/School of Basic Medical Sciences/Library/Key Laboratory for Quality of Salt Alkali Resistant TCM of Hebei Administration of TCM, North China University of Science and Technology, Tangshan 063210, China; School of Life Sciences/School of Basic Medical Sciences/Library/Key Laboratory for Quality of Salt Alkali Resistant TCM of Hebei Administration of TCM, North China University of Science and Technology, Tangshan 063210, China; School of Life Sciences/School of Basic Medical Sciences/Library/Key Laboratory for Quality of Salt Alkali Resistant TCM of Hebei Administration of TCM, North China University of Science and Technology, Tangshan 063210, China; State Key Laboratory of Vegetable Biobreeding, Beijing Vegetable Research Center, Beijing Academy of Agriculture and Forestry Science, Beijing 100097, China; School of Life Sciences/School of Basic Medical Sciences/Library/Key Laboratory for Quality of Salt Alkali Resistant TCM of Hebei Administration of TCM, North China University of Science and Technology, Tangshan 063210, China; School of Life Sciences/School of Basic Medical Sciences/Library/Key Laboratory for Quality of Salt Alkali Resistant TCM of Hebei Administration of TCM, North China University of Science and Technology, Tangshan 063210, China; School of Life Sciences/School of Basic Medical Sciences/Library/Key Laboratory for Quality of Salt Alkali Resistant TCM of Hebei Administration of TCM, North China University of Science and Technology, Tangshan 063210, China; School of Life Sciences/School of Basic Medical Sciences/Library/Key Laboratory for Quality of Salt Alkali Resistant TCM of Hebei Administration of TCM, North China University of Science and Technology, Tangshan 063210, China; School of Life Sciences/School of Basic Medical Sciences/Library/Key Laboratory for Quality of Salt Alkali Resistant TCM of Hebei Administration of TCM, North China University of Science and Technology, Tangshan 063210, China; School of Life Sciences/School of Basic Medical Sciences/Library/Key Laboratory for Quality of Salt Alkali Resistant TCM of Hebei Administration of TCM, North China University of Science and Technology, Tangshan 063210, China; School of Life Sciences/School of Basic Medical Sciences/Library/Key Laboratory for Quality of Salt Alkali Resistant TCM of Hebei Administration of TCM, North China University of Science and Technology, Tangshan 063210, China; School of Life Sciences/School of Basic Medical Sciences/Library/Key Laboratory for Quality of Salt Alkali Resistant TCM of Hebei Administration of TCM, North China University of Science and Technology, Tangshan 063210, China; National Center of Technology Innovation for Comprehensive Utilization of Saline-Alkali Land, Dongying 257300, China

## Abstract

Since its inaugural release in 2022, The Vegetables Information Resources (TVIR) has been a cornerstone for genomics and genetic breeding studies within the vegetable research community. With advancements in sequencing technologies leading to an influx of new genome sequences, TVIR has been upgraded to version 2.0 (http://tvir2.bio2db.com/), expanding from 59 to 84 vegetable species and introducing new functional modules to accelerate research. This upgrade incorporates a CRISPR/Cas9 resource module, which integrates four specialized tools: CasFinder, CasOT, Crisflash, and CRISPRCasFinder, to facilitate gene editing research. The database further features dynamic synteny analysis with an interactive interface, enabling users to visualize genomic relationships between species. Additionally, two novel bioinformatics tools Hmmsearch and CRISPRCasViewer are integrated to enhance comparative and functional genomic analyses. TVIR 2.0 retains all TVIR 1.0 features while updating resistance gene identification, expanding from 3 to 8 types, and transcription factor datasets, now including 237 431 TFs, an increase from 172 493.The database integrates comprehensive genomic, transcriptomic, and functional annotation data, providing freely accessible resources for vegetable breeding and gene editing.

## Introduction

The Vegetables Information Resources (TVIR) is the first comprehensive database for vegetable genomics sharing and analysis. It has been released for the first time since 2022 and provides abundant data resources for vegetable genomics and genetic breeding research [1]. TVIR collected vegetable genome resources from several major public or private databases, such as NCBI, JGI, BRAD, and TBGR [2, 3]. Its initial version contains gene annotation and detection of the main functional genes of 59 vegetable crops. In addition, TVIR 1.0 also provided four bioinformatics analysis tools, which provided effective tool support for the analysis and verification of gene function in the vegetable crops.

In the past 2 years, with the rapid advancements in sequencing technology and the reduction of sequencing cost, many high-quality reference genome sequences of vegetables have been sequenced or upgraded [4]. For example, the allotetraploid horseradish (*Armoracia rusticana*) has obtained a complete chromosome-level telomere-to-telomere (T2T) gapless reference genome through a variety of sequencing strategies [5]. Some vegetable crops with important nutritional values and research significance have also been sequenced for the first time, such as *Allium fistulosum* [6], *Glebionis coronaria* [7], and *Cichorium endivia* [8]. Their genomic resources have been reported in their respective literature or scattered in different databases. Therefore, there is an urgent need for us to constantly update TVIR to make it more comprehensive.

TVIR 2.0 includes a total of 84 vegetables, all of which can collect complete genomic data. To explore the gene editing research of vegetables, TVIR 2.0 also provides a new module CRISPR/Cas9 resource, which includes the use of four popular analysis tools CasFinder, CasOT, Crisflash, and CRISPRCasFinder to identify and predict vegetable crop candidate sequences, providing multiple resources to facilitate scientific research. To clarify the situation of gene duplication or loss after whole-genome duplication (WGD) or whole-genome triplication (WGT) events, TVIR 1.0 provides syntenic analysis between various vegetable crops. However, it only provided static pdf resources at that time. To allow users to browse the relationship between vegetables more clearly and accurately, we have now upgraded this module and constructed a user-friendly and dynamic interactive interface, hoping to provide researchers with a more convenient and effective operating platform. In addition, two toolkits Hmmsearch and CRISPRCasViewer were introduced. These tools provide important value for vegetable genomics, genetic breeding and gene editing research. The newly updated TVIR 2.0 can be accessed online through http://tvir2.bio2db.com/.

**Figure 1:**
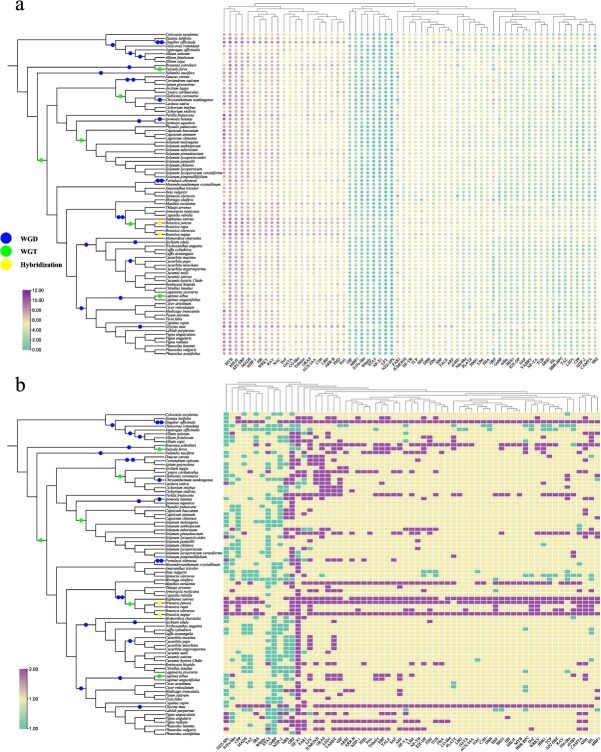
Transcription factors (TFs) family mapping of 84 vegetable crops. (a) Circle diagram shows the number of each TF family in each species. The number of each TF were converted by log2. (b) In each vegetable, the ratio of the number of each TF family to the number of members of *Arabidopsis thaliana*.

**Figure 2:**
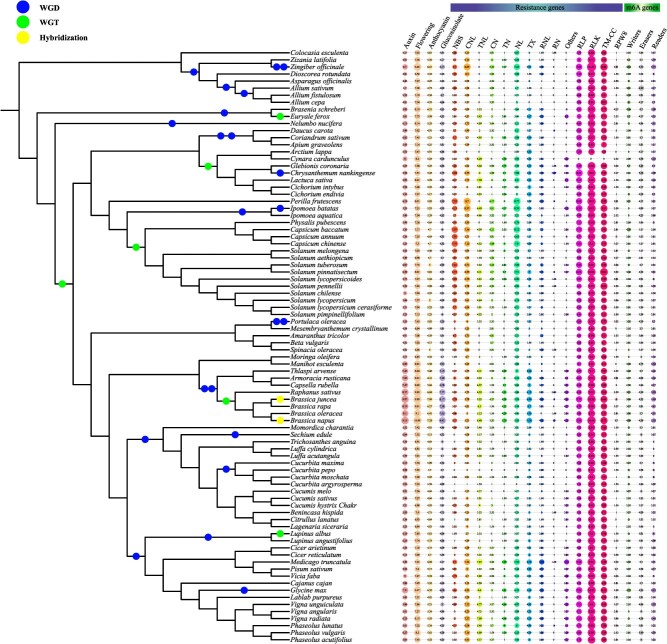
Plot of number of members of six functional gene families, including auxin, anthocyanin, flowering, glcosinolte, resistance, and m6A genes, in the 84 vegetable crops. The number of each gene families were converted by log2.

**Figure 3:**
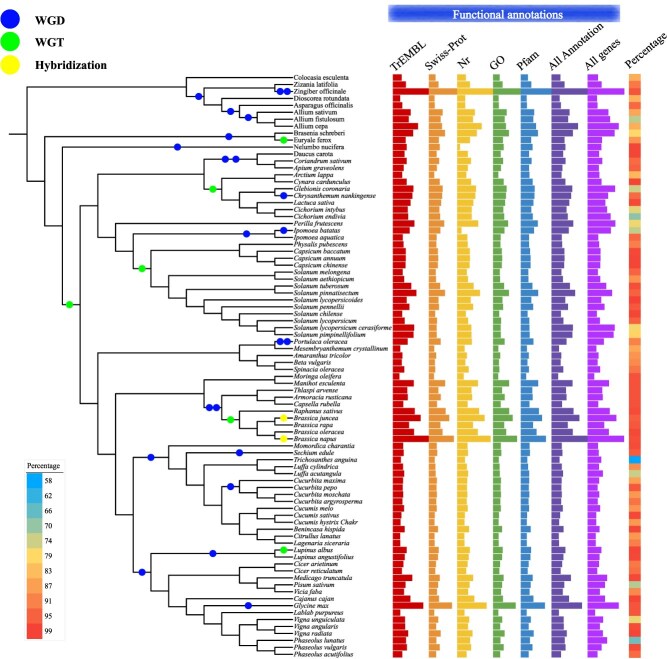
Bar plots of the number of gene functional annotations in the 84 vegetable crops.

**Figure 4:**
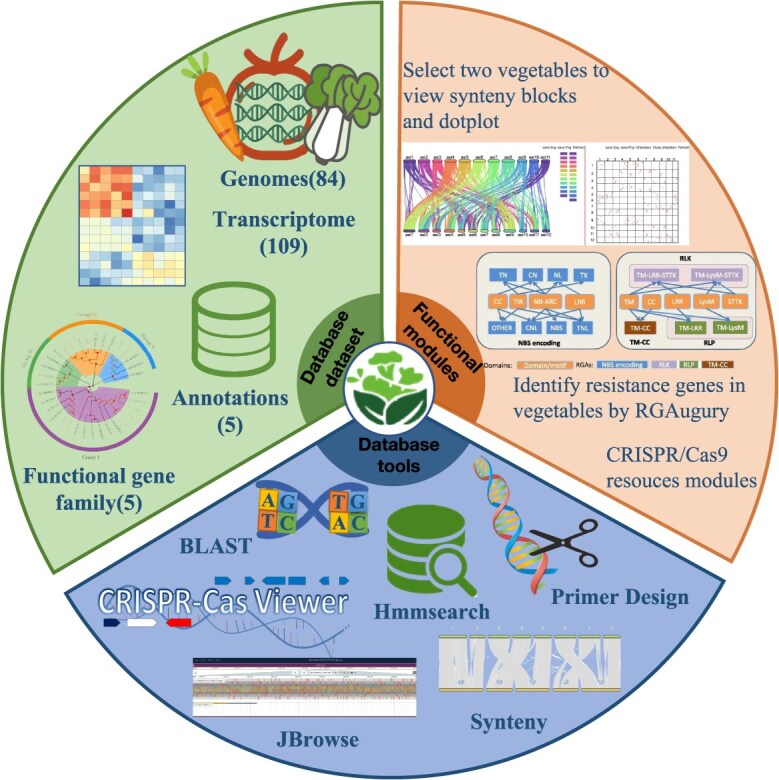
The Molecular Mechanism of CRISPR/Cas9 System. This image is drawn by Figdraw, and the export ID is IOPIR64454.

## Results

### Database dataset update

**Figure 5:**
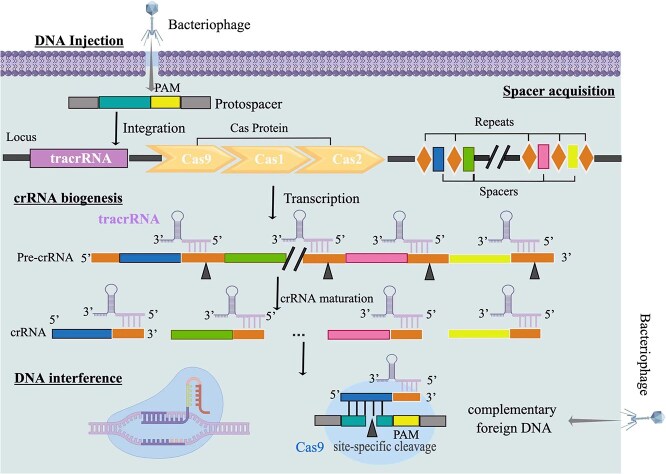
Architecture and genome updates of TVIR 2.0. In the upper left corner, it represents data architecture of the updated TVIR. The digit in brackets indicate the number of total datasets. In the upper right corner, the function module of TVIR update is represented. In the bottom of the figure, it represents the current analysis tool in the database.

TVIR 2.0 is a comprehensive vegetable genomics resource database based on Django and Mysql framework. Compared with the previous version, the dataset mainly includes 84 vegetable genomes, 110 vegetable transcriptome information, 237 431 transcription factors (Fig. 1; Tables S4 and S5), syntenic genes and homologous genes between 84 vegetable genomes, and 33 019 genes from four functional gene families (Auxin, Anthocyanin, and Flowering, and Glucosinolate) (Fig. 2; Table S6). In addition, a comprehensive gene function annotation consisting of Swiss-Prot and TrEMBL of the UniProt knowledgebase (https://www.uniprot.org) [9], Pfam (v34.0) (http://pfam.xfam.org) [10], Gene Ontology (GO, http://geneontology.org) [11], the non-redundant protein sequence database (Nr, https://www.ncbi.nlm.nih.gov) (Fig. 3; Table S2), CRISPR sequences, Cas proteins, gRNAs, target genes, and potential off-target sites of 84 vegetable genomes (Fig. 4; Table S3). Among them, to facilitate the use of researchers, we have updated the identification method of resistance genes, and changed the types of resistance genes from three to eight (Fig. 5; Table 1; Table S7). TVIR 2.0 also designs and updates a variety of practical and flexible tools for vegetable crop genomics and breeding, including BLAST, Primer design [12, 13], Synteny [14], Jbrowse [15], Hmmsearch [16], and CRISPRCasviewer. All datasets can be downloaded and analyzed for free in TVIR 2.0.

### Transcription factors dataset update

Transcription factors play a key role in various stresses and plant development [17]. TVIR 2.0 dataset increased from 172 493 in the previous version to 237 431 transcription factors (Fig. 1a). The four TF families with the largest number of genes are *MYB* (25960), *NBS* (20052), *AP2/ERF* (16826) and *bHLH* (14651). Compared with *Arabidopsis thaliana*, the *NF-X1* gene family was significantly expanded, while *GeBP*, *bZIP-2*, and *STAT* gene family contracted in most vegetable species (Fig. 1b).

### Functional gene dataset update

Several important agronomic-related functional gene families were identified in 84 vegetables, including 6547 auxin genes, 3451 anthocyanin genes, 19 723 flowering genes and 3298 glucosinolate genes (Fig. 2). All these functional genes play an important role in the breeding and research of vegetable varieties. Users can quickly search and download related genes directly from the database.

### Gene annotation dataset update

The gene annotation information of each vegetable crop was identified based on five protein databases (GO, Nr, TrEMBL, SwissProt, and Pfam). The vegetable with the least gene annotation number was *Trichosanthes anguina*, which was annotated to 58.29% of the genes, and the vegetable with the most gene annotation was *Benincasa hispida*, which was annotated to 99.88% of the genes. The number of annotated genes of 84 vegetable crops was plotted using the ITOL website (https://itol.embl.de) (Fig. 3).

### Addition of new functional modules

In TVIR 2.0, there are two main update function modules, including synteny and dotplots interaction and CRISPR/Cas9 resources modules.

#### Synteny and dotplots interaction

To facilitate researchers to explore syntenic genes between vegetable crop genomes, TVIR 2.0 changed the previous version of static syntenic search, using Python scripts to update the new dynamic synteny and dotplots interaction module (Fig. 5). TVIR 2.0 allows users to easily select any two vegetables, and then view their genomic syntenic blocks and interspecies dotplots in the database. You can click on the block of interest to obtain its syntenic genes, and the syntenic maps and dotplots drawn can also be downloaded and saved by PNG and SVG.

#### CRISPR/Cas9 resources modules

**Figure 6:**
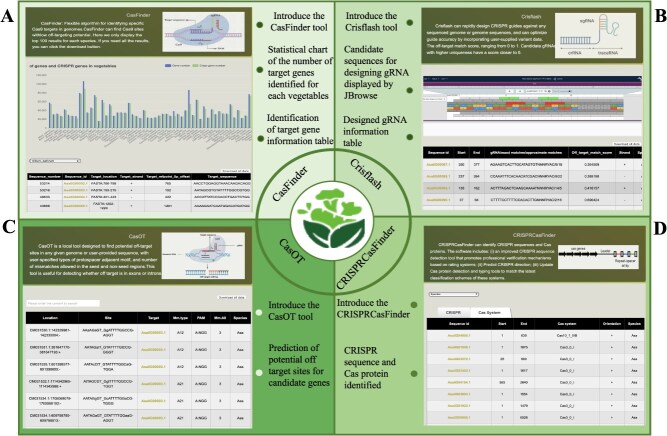
CRISPR/Cas9 resources modules in TVIR.

The CRISPR/Cas9 system includes the CRISPR sequence (Clustered Regularly Interspaced Short Palindromic Repeats) and the adjacent Cas protein (CRISPR-associated) [18, 19]. The system can accurately identify and cut off exogenous nucleic acids, silence the expression of exogenous genes, and maintain the stability of its own genetic system. It is a defense method for prokaryotes to resist the invasion of exogenous genetic materials (such as phages and exogenous plasmids) (Fig. 4). Based on this precise targeting function, RNA can accurately locate different gene targets through simple design, so it can efficiently edit and detect target genes, showing great application prospects (Fig. 6) [36–24]. We employed four distinct tools to comprehensively profile the genomes and screen for candidate sequences of 84 vegetable crops. Among these, the CasFinder tool was utilized to systematically identify 487 857 crop-specific CRISPR guide sequences, demonstrating its efficacy in pinpointing precise genomic targets for gene editing applications (Table S3). The CasOT tool was used to detect the possible off-target sites of each guide sequence. The study of off-target effect can provide abundant data resources for experimental personnel, which is convenient for further optimizing the design of sgRNA and improving editing efficiency [25]. In addition, we also designed gRNAs for 84 vegetable candidate genes. Crisflash software is more than an order of magnitude faster than similar tools, which can effectively and robustly score the potential off-target of all possible candidate CRISPR-guided oligonucleotides even using a single CPU core. The CRISPRCasFinder tool was also used to identify 84 CRISPR sequences and adjacent Cas proteins in vegetables (Fig. 6). TVIR hopes to provide data resources for genetic breeding and gene editing design of vegetable crops through these resources [26–28].

### New additions to tools

#### Hmmsearch

HMMER is a very powerful software package for biological sequence analysis based on hidden Markov model. Its general purpose is to identify homologous protein or nucleotide sequences and sequence alignment [29]. Compared with BLAST, FASTA and other sequence alignment and database search tools, HMMER is more accurate. Therefore, we add the Hmmsearch tool to TVIR 2.0. Users can enter different HMM model numbers or upload HMM files for comparison and analysis to obtain a list of the sequences that best match the configuration file. The resulting file can be downloaded and viewed immediately.

#### CRISPRCasViewer

To make users better understand the CRISPR sequence and Cas protein of vegetable crops, TVIR 2.0 add the tool that can view the results of the CRISPRCasFinder program. There are three ways to visually display the generated results: Linear view, Circular view, and Scatter plot view. Users submit the json file generated by CRISPRCasFinder and select the genes of interest for visual display.

**Table 1:** The comparisons on dataset volume, update functional module, and tools between version 1.0 and 2.0 of TVIR database

**TVIR**			**v1.0**	**v2.0**
	Species		59	84
	Transcriptome		97	109
	TF genes		172 493	237 431
	Functional genes	Auxin genes	5215	6547
		Anthocyanin genes	2437	3451
		Flowering genes	15 002	19 723
		Glucosinolate genes	2639	3298
**Dataset volume**	Types of resistance genes		3	8
	CRISPR correlation analysis	Identify target genes	No	Yes
		Identify off-target sites	No	Yes
		Design gRNAs	No	Yes
		Identify CRISPR sequence and Cas protein	No	Yes
**Update functional module**	Synteny analysis		Static	Dynamic
	Blast		Yes	Yes
	Synteny		Yes	Yes
	Primer Design		Yes	Yes
	JBrowse		Yes	Yes
	Hmmsearch		No	Yes
**Tools**	CRISPRCasViewer		No	Yes

## Discussion

Vegetables, integral to human nutrition, hold significant value for research. TVIR 2.0, with its enriched dataset and user interface, continues to be a vital resource [30–32]. The user interface of TVIR 2.0 has been continuously optimized. The collection of vegetable genomes has also increased from 59 to 84, and the transcriptome information has also increased from 98 to 110. On the basis of retaining the main functions of the previous version, we also updated the two main functional modules Dynamic synteny and dotplots and CRISPR/Cas9 resources module. The updates to the synteny analysis and CRISPR/Cas9 resources module are designed to offer smarter services to the scientific community. Synteny analysis allows users to easily select any two vegetables, draw their genomic syntenic blocks and interspecies dotplots online, and click on the block of interest to obtain its syntenic genes. The CRISPR/Cas9 resource module uses four types of tools to identify the target genes, CRISPR sequences, and Cas proteins of vegetables. Potential off-target sites were predicted, and gRNAs were designed and evaluated for vegetables. The addition of these functional modules aims to provide researchers with better and smarter services. In addition, TVIR 2.0 also introduced two new tools, Hmmsearch and CRISPRCasViewer, to provide researchers with a wealth of online tools.

Notably, two limitations should be acknowledged. One is that genome assemblies for certain species in the database remain at the scaffold level, leading to incomplete functional gene annotation. Fragmented assemblies hinder comprehensive gene prediction, as seen in *Trichosanthes anguina*, where only 58.29% of genes were annotated across five databases. Another is that CRISPR off-target predictions by CasOT and Crisflash require experimental validation, as in silico scores may overestimate editing specificity. In TVIR 2.0, 18.3% of predicted off-target sites in *Brassica rapa* lacked experimental confirmation.

With ongoing genomic advancements, TVIR 2.0 will integrate single-cell omics and pan-genome data to address current assembly biases. Future updates will also benchmark tool accuracy against BRAD and PlantGIR [33], while expanding CRISPR validation workflows to experimental validation.

## Materials and methods

### Data collection and processing

TVIR 2.0 continues with the methodology established in the previous version, extracting genomic data from plaBiPD (https://www.plaBiPD.de/index.ep) and major databases [1]. First, we extracted the reported vegetable genomic information from plaBiPD, and then collected the genome sequences, general feature format (GFF) files, coding DNA sequence (CDS), and protein sequence in the major public or personal databases. Spliced genes were deleted to avoid redundant sequences using a custom Perl script. To standardize data formats for downstream analysis, a custom Python script was employed to convert GFF files into a unified five-column format, including chromosome, gene, start position, end position, and strand. This format served as the foundation for subsequent analyses, ensuring consistency across 84 vegetable genomes. Compared with the previous version, we added new genomic information of 25 vegetable crops, complete with detailed classifications and annotations (Table S1).

### Database dataset update

Identifying the transcription factor family of vegetable crops through the PlantTFDB (https://planttfdb.gao-lab.org), using Hidden Markov Model (HMM) of transcription factor family [34–36]. The pfam_scan.pl program and pfam database (v34.0) were used to determine the domain types of all proteins. The identification method for functional gene families and gene annotation is the same as that of TVIR 1.0 [1]. The database construction according to the previous reports [33, 37–39].

### Identification of resistance genes

The Pfam and Blastp were used to identify three type resistance (R) genes, including NBS, RLK and RLP family genes. To facilitate the study of resistance genes, TVIR 2.0 uses RGAugury (https://bitbucket.org/yaanlpc/rgaugury/src/master/) to identify resistance genes in the vegetable genomes. Resistance gene analogs (RGAs) include proteins encoding NBS, receptor-like protein kinases (RLKs) and receptor-like proteins (RLPs) [40–42]. The resistance gene of 84 vegetable genomes was identified from eight domains, and these results can be obtained by searching on TVIR 2.0.

### CRISPR/Cas9 module related analysis

#### Identification of CRISPR target genes

Firstly, the RepeatMasker (v4.1.10, http://www.repeatmasker.org) program was used to shield the repetitive sequences of the genomes of 84 vegetable species, and then the Bowtie (v2.0, https://bowtie-bio.sourceforge.net/bowtie2/index.shtml) was used to create an index for each genome [43]. The CasValue_v2.pl and CasFinder.pl scripts in the CasFinder (v1.0, https://arep.med.harvard.edu/CasFinder/) were used to design the guide sequence of the CRISPR sequence, and perl program was used to filter the candidate sequence to obtain the target genes of the CRISPR for each vegetable genome [44].

#### Prediction of potential off target sites for CRISPR

The off-target sites were identified by CasOT software [45] (v1.0, http://eendb.zfgenetics.org/casot/). The maximum number of mismatches allowed in the seed region of potential was set to 2, and the maximum number of mismatches allowed in the non-seed region of was set to 2. The off-target site information of each sequence can be obtained through the TVIR 2.0 database.

#### Design and evaluation of gRNAs

TVIR 2.0 uses Crisflash (https://github.com/crisflash/crisflash/) software to design gRNAs, which can rapidly design gRNAs against any genome sequences, and can optimize guide accuracy by incorporating user-supplied variant data [46]. The off-target match score, ranging from 0 to 1. Candidate gRNAs with higher uniqueness have a score closer to 0. However, TVIR 1.0 only shows each species Candidate gRNAs with highest uniqueness by the perl program.

#### Identification of CRISPR sequence and Cas protein

We used CRISPRCasFinder (https://github.com/dcouvin/CRISPRCasFinder) to identify CRISPR sequences and Cas proteins. CRISPR consists of short highly conserved repeats and different spacers [47]. Repeat mostly has palindrome structure. Spacer is homologous to exogenous DNA (such as plasmids or viruses) [48]. We identified CRISPR sequences and Cas proteins in 84 vegetable genomes and provided json files generated by the results, which can also be visualized by online tool CRISPRCasViewer.

## Consent for publication

All the authors approved the manuscript and consented to publication of the work.

## Supplementary Material

Web_Material_uhaf239

## Data Availability

TVIR 2.0 is freely available to the public without any registration or login requirements (http://tvir2.bio2db.com/). All the related datasets can be downloaded from the database.
